# Charge density wave transition in single-layer titanium diselenide

**DOI:** 10.1038/ncomms9943

**Published:** 2015-11-16

**Authors:** P Chen, Y. -H. Chan, X. -Y. Fang, Y Zhang, M Y Chou, S. -K. Mo, Z Hussain, A. -V. Fedorov, T. -C. Chiang

**Affiliations:** 1 Department of Physics, University of Illinois at Urbana-Champaign, 1110 West Green Street, Urbana, Illinois 61801-3080, USA; 2 Frederick Seitz Materials Research Laboratory, University of Illinois at Urbana-Champaign, 104 South Goodwin Avenue, Urbana, Illinois 61801-2902, USA; 3 Advanced Light Source, Lawrence Berkeley National Laboratory, Berkeley, California 94720, USA; 4 Institute of Atomic and Molecular Sciences, Academia Sinica, Taipei 10617, Taiwan; 5 National Laboratory of Solid State Microstructures, School of Physics, Collaborative Innovation Center of Advanced Microstructures, Nanjing University, Nanjing 210093, China; 6 Stanford Institute of Materials and Energy Sciences, SLAC National Accelerator Laboratory, Menlo Park, California 94025, USA; 7 School of Physics, Georgia Institute of Technology, Atlanta, Georgia 30332, USA; 8 Department of Physics, National Taiwan University, Taipei 10617, Taiwan

## Abstract

A single molecular layer of titanium diselenide (TiSe_2_) is a promising material for advanced electronics beyond graphene—a strong focus of current research. Such molecular layers are at the quantum limit of device miniaturization and can show enhanced electronic effects not realizable in thick films. We show that single-layer TiSe_2_ exhibits a charge density wave (CDW) transition at critical temperature *T*_C_=232±5 K, which is higher than the bulk *T*_C_=200±5 K. Angle-resolved photoemission spectroscopy measurements reveal a small absolute bandgap at room temperature, which grows wider with decreasing temperature *T* below *T*_C_ in conjunction with the emergence of (2 × 2) ordering. The results are rationalized in terms of first-principles calculations, symmetry breaking and phonon entropy effects. The observed Bardeen-Cooper-Schrieffer (BCS) behaviour of the gap implies a mean-field CDW order in the single layer and an anisotropic CDW order in the bulk.

## Abstract (web summary)

Single molecular layers of TiSe_2_ are promising for advanced electronic applications, and it is therefore important to characterize their phases. Here, the authors use ARPES to detect a charge density wave transition without Fermi surface nesting and that takes place at a temperature higher than in bulk.

## Graphical abstract

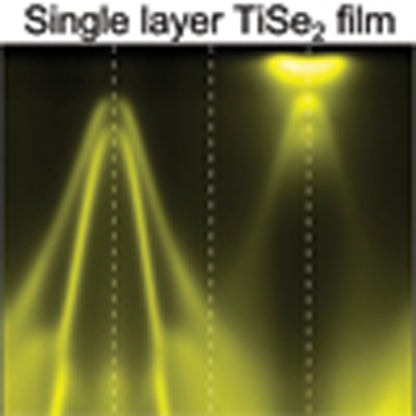

Titanium diselenide (TiSe_2_) is a member of a vast family of transitional metal dichalcogenides, many of which show charge density wave (CDW) transitions at low temperatures leading to periodic modulations of the electronic charge density. The resulting superlattices can be either commensurate or incommensurate. The CDW order can compete with other transitions such as superconductivity and antiferromagnetism, and it is a phenomenon of great interest in solid state physics^1^,^2^,^3^. Specifically, TiSe_2_, with a simple (2 × 2 × 2) CDW transition at 200 K in the bulk^4^, remains an intensely debated case^5^,^6^,^7^,^8^,^9^,^10^. The transition has been attributed variably to excitonic interaction, band-type Jahn–Teller effects, and so on^5^,^11^,^12^. A detailed investigation of the electronic structure is complicated by the three-dimensional nature of the CDW order. The perpendicular electronic momentum is not necessarily conserved in angle-resolved photoemission spectroscopy (ARPES) measurements, making it difficult to pinpoint the gap locations in the Brillouin zone. A single layer of TiSe_2_, by contrast, has a two-dimensional (2D) electronic band structure, and the gap of interest is limited to the one bridging the 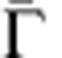 and 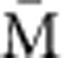 points in the Brillouin zone. A recent study by scanning tunneling microscopy of a single layer of TiSe_2_ revealed a (1 × 1) structure at room temperature and a (2 × 2) superstructure at low temperatures, but it offered no information otherwise on the nature and details of the CDW transition^13^. A detailed mapping of the electronic structure of the single-layer case will not only help resolve the issues related to the bulk transition, but also reveal the relevant CDW physics at the 2D limit. A broader impetus for our work is the search and discovery of suitable molecular layers for advanced electronics with minimal physical dimensions suitable for integration and easily amenable to quantum engineering.

In this work, we employ molecular beam epitaxy to prepare high-quality single-layer TiSe_2_ on a suitably chosen substrate with a rather inert surface to minimize the substrate effects on the overlayer. High-resolution ARPES measurements as a function of temperature reveal intricate details including gap evolution and band folding in connection with the (2 × 2) CDW ordering. Surprisingly, the measured transition temperature in the single layer is substantially higher than the bulk transition temperature. We explain these results with the aid of first-principles calculations. The observed temperature-tunable gap in the single-layer case indicates that the system is indeed a strong candidate for applications.

## Results

### Film structure and electron diffraction patterns

**Figure 1:**
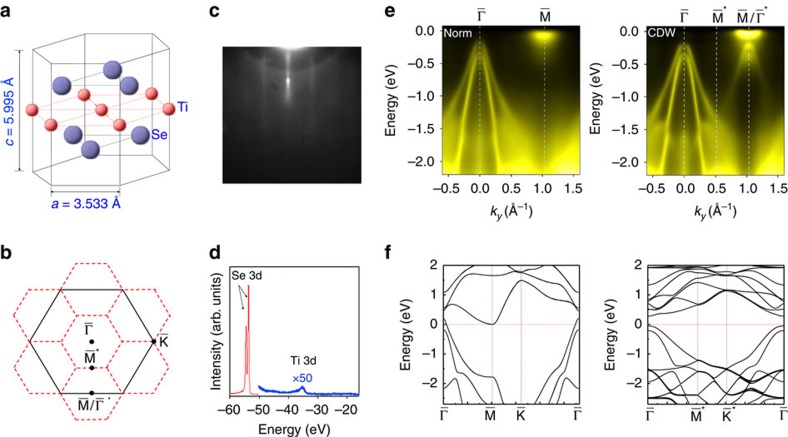
Film structure and electronic bands. (**a**) Atomic structure of a single layer of TiSe_2_. In bulk TiSe_2_ the layer spacing is *c* as indicated. (**b**) Brillouin zones of the (1 × 1) and (2 × 2) structure outlined in black and red, respectively. (**c**) A reflection high-energy electron diffraction pattern after film growth. (**d**) Core-level scans taken with 100 eV photons. (**e**) ARPES maps taken from a single layer of TiSe_2_ along the 
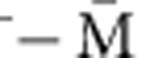
 direction for the (1 × 1) normal phase at room temperature and the (2 × 2) CDW phase at 10 K. All data were taken with 58 eV photons. (**f**) Calculated DFT band structure of the (1 × 1) and (2 × 2) phases with the HSE hybrid functional.

Our single layers of TiSe_2_ were grown *in situ* on a bilayer-graphene-terminated 6H-SiC (0001) (refs 14, 15). The interfacial interaction is expected to be of the van der Waals type, resulting in a nearly decoupled TiSe_2_ overlayer. The crystal structure (Fig. 1a) consists of a hexagonal planar net of Ti atoms sandwiched between two Se atomic layers. The (1 × 1) and (2 × 2) Brillouin zones are also hexagonal (Fig. 1b). Reflection high-energy electron diffraction (Fig. 1c) reveals a well-ordered layer with the same in-plane lattice constant as that of bulk TiSe_2_ within our experimental accuracy. Scans of the core levels (Fig. 1d) show a much stronger Se 3*d* signal than the Ti 3*d* signal partly because the top atomic layer is made of Se atoms.

### ARPES spectra and calculated band structure

**Figure 2:**
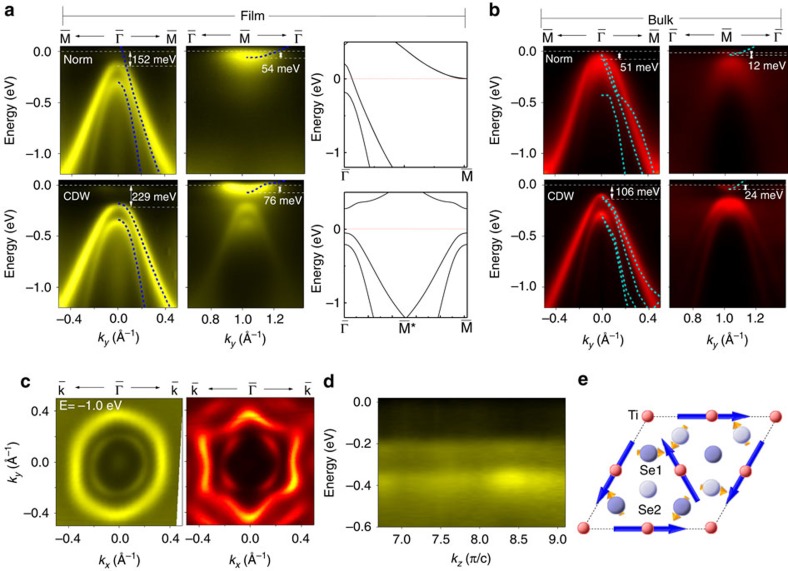
Bandgaps in single-layer film and bulk TiSe_2_. (**a**) Comparison of ARPES spectra and calculated bands (blue dashed curves) for single-layer TiSe_2_ in both the normal and CDW phases. The data were taken with 58 eV photons. (**b**) Comparison of ARPES spectra and calculated bands (cyan dashed curves) for bulk TiSe_2_. The photon energies used were 58, 61, 67 and 61 eV for the top left, top right, bottom left and bottom right panels, respectively. (**c**) Constant-energy-contour maps around 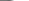 for film (yellow-colour-coded) and bulk (red-colour-coded) at energy of −1.0 eV, taken with 58 eV photons. (**d**) Dispersions of the valence band maxima of single-layer TiSe_2_ as a function of *k*_*z*_ (or photon energy). (**e**) Atom displacements in the CDW phase. Se1 and Se2 correspond to Se atoms in the top and bottom layers, respectively. The length of each arrow indicates the magnitude of the displacement amplified by 50 times.

ARPES maps taken from the single-layer sample along the 
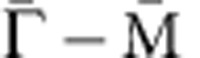
 direction (Fig. 1e) for both the (1 × 1) normal phase at room temperature and the (2 × 2) CDW phase at 10 K are compared with the (1 × 1) and (2 × 2) band structure (Fig. 1f) deduced from first-principles calculations based on density functional theory using the Heyd–Scuseria–Ernzerhof (HSE) hybrid functional (Methods section). In the normal phase, the band structure near the Fermi level consists of a pair of concave bands centred at the 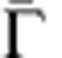 point that are primarily derived from the Se 4*p* states. At the 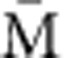 point, the bottom of a convex band reaches just below the Fermi level, and this band is primarily derived from the Ti 3*d* states. The top of the Se 4*p* band and the bottom of the Ti 3*d* band are separated by a gap of 98 meV at room temperature. The calculation with the HSE functional including the spin–orbit interaction yields instead a negative gap of 0.20 eV. Further calculations with the *GW* approximation within a many-body perturbation theory reduce the negative gap to 0.18 eV at the *G*_0_*W*_0_ level and to 0.08 eV with a self-consistent *G*. The calculation assumes *T*=0, while the (1 × 1) structure is observed only at high temperatures. For the (2 × 2) CDW phase, the ARPES features become sharper because of the lower sample temperature. The Se 4*p* bands are now repeated at the 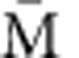 point, which is also the zone centre 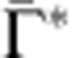 after (2 × 2) zone folding. The folded (or umklapp) Se bands at 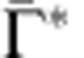 have a lower intensity as expected because the superlattice distortion is weak. Experimentally, the gap is 153 meV at 10 K, which is substantially larger than the room-temperature gap value of 98 meV. For comparison, the calculated HSE bandgap for an optimized structure at *T*=0 is 330 meV. The calculated total energy per chemical unit is lower by 5 meV (4 meV) for the CDW phase relative to the normal phase in the single layer (bulk). The calculated atomic displacements in the CDW phase are 0.09 Å for Ti and 0.03 Å for Se, respectively, as shown in Fig. 2e.

A detailed comparison of the bands near the Fermi level between experiment and theory is presented in Fig. 2a for single-layer TiSe_2_, where the calculated bands are shown as blue dashed curves and are aligned in energy by matching the Ti 3*d* band bottom or Se 4*p* band top where appropriate. The corresponding results for bulk TiSe_2_ (Fig. 2b) in both the normal and CDW phases reveal similar behaviour: a small positive gap for the normal phase becomes larger for the CDW phase. One notable difference is that folding of the Se 4*p* bands is evident for the normal phase of the bulk crystal, which has been reported before and attributed to fluctuation effects^16^. No such folded bands are observed for the single-layer sample in the normal phase. Constant-energy ARPES contours (Fig. 2c) at energy of −1.0 eV for the bulk and single-layer samples in the CDW phase are similar because the bulk material is quasi-2D, but there are also clear differences. The pronounced warping for the bulk case can be attributed to interlayer coupling effects. We have performed band mapping along *k*_*z*_ over a wide range by scanning the incident photon energy over 30–80 eV for the ARPES measurements of the single-layer sample. The results for the Se 4*p* states at the 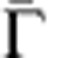 point in the CDW phase (Fig. 2d) show no detectable energy dispersion, in agreement with the 2D nature of the system.

### Temperature dependence of the bandgap

**Figure 3:**
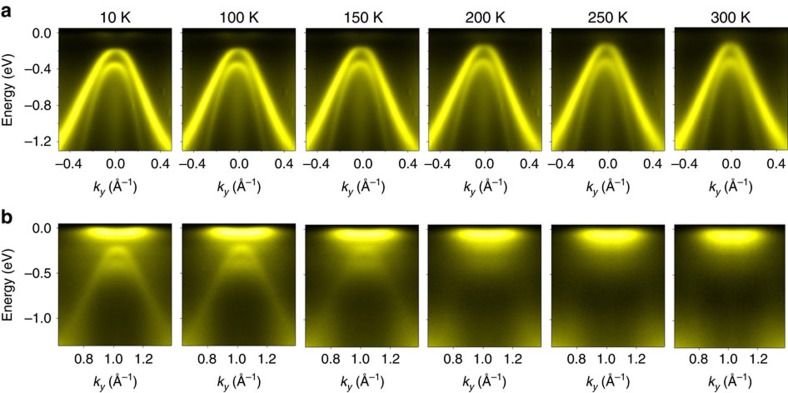
Temperature dependence of the band structure. (**a**) ARPES spectra around the zone centre 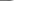 reveal that the valence band top shifts towards the Fermi level when the temperature is increased from 10 to 300 K. (**b**) ARPES spectra around the zone boundary 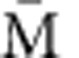. Emergence of the back-folded valence bands at low temperature indicates formation of the (2 × 2) CDW phase. All data were taken with 58 eV photons.

**Figure 4:**
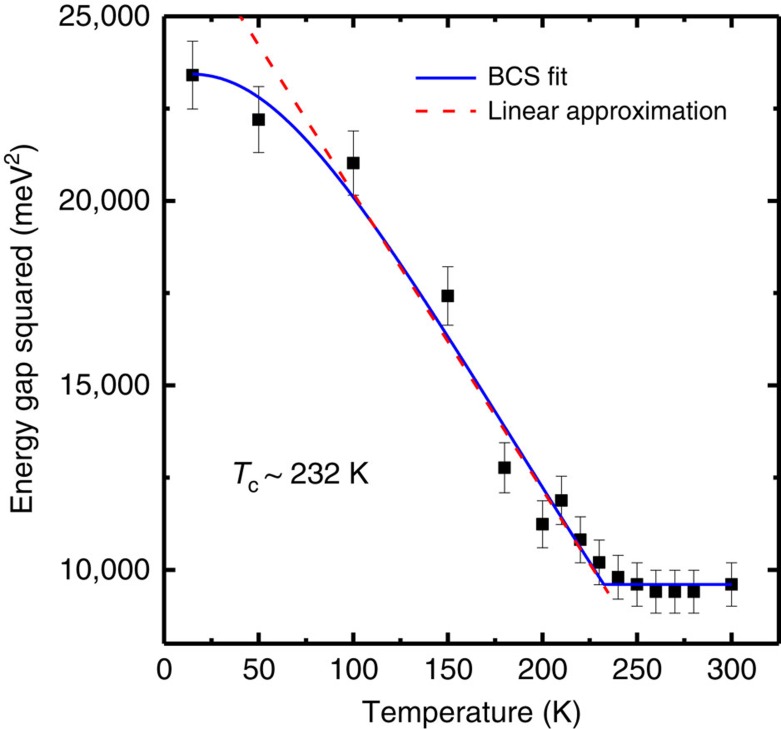
BCS-like behaviour of the bandgap evolution with temperature. The measured gap squared is shown as squares. The blue curve is a fit using a BCS-type gap equation. The red-dashed line is a linear approximation of the data near but below the transition temperature *T*_C_. The error bars represent the s.d. of the band position from fitting to the band dispersion at each temperature.

A temperature scan of the bands near 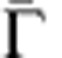 and 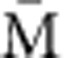 (Fig. 3) reveals variations of the band positions and the intensities of the folded bands. The energy gap is extracted from the data, and the square of the energy gap, plotted as a function of *T* (Fig. 4), shows that the gap becomes smaller as *T* increases and saturates to a constant value above a transition temperature *T*_C_=232 K. It is interesting to note that the square of the energy gap follows closely a linear behaviour for *T* near but below *T*_C_, as indicated by the red-dashed line:


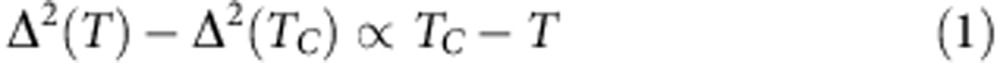


which suggests a mean-field behaviour. The blue solid curve is a fit to the data using a semi-phenomenological BCS gap equation based on the mean-field theory:


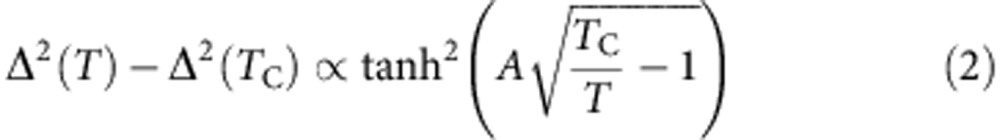


where *A*=1.16 is a proportional constant and *T*_C_=232±5 K. Equation (2) reduces to equation (1) for *T* near *T*_C_. The observed mean-field behaviour is not surprising because quantum fluctuation effects are negligible compared with thermal fluctuation effects considering the high *T*_C_ value for this system.

## Discussion

CDW transitions are often attributed to Fermi surface nesting, but with the existence of a bandgap in both the normal and CDW phases there is no nesting in the present case. Our first-principles calculations show that the gap widens with an increasing (2 × 2) distortion because of the lifting of the conduction band degeneracy that couples indirectly to the valance bands^17^. The larger gap pushes the occupied Se 4*p* states to lower energies, resulting in total energy-lowering initially, but this process is soon counteracted by other effects such as an increase in elastic energy. The calculated Ti displacement in the CDW phase of monolayer TiSe_2_ is about 2.5% of the calculated lattice constant (*a*=3.538 Å), which is very close to the value of 2.4% in the bulk as measured by neutron scattering^10^. The fact that our HSE calculations show the (2 × 2) phase to be the ground state at *T*=0 indicates that the CDW phase is entirely a band structure effect. There is no need to invoke additional or exotic many-body interactions beyond the HSE exchange and correlation effects.

The question is then what drives the system into the (1 × 1) structure at higher temperatures. Like the Jahn–Teller and ferromagnetic transitions, CDW transitions can be described as a result of spontaneous symmetry breaking. For TiSe_2_, the transition involves atomic displacements that can be spatially reversed to yield a configuration with the same total energy but separated from the original configuration by an energy barrier. At low temperatures, the system is frozen in one of the two configurations. At higher temperatures, thermal effects (or phonon entropy effects) allow the system to fluctuate between the two configurations, resulting in average zero atomic displacements or a (1 × 1) structure on average. The physics is very similar for the different types of phase transitions, and indeed, the same mean-field behaviour is observed for the single-layer TiSe_2_.

The enhanced *T*_C_ for the single-layer TiSe_2_ relative to the bulk indicates that the CDW phase in the single layer is more robust^18^. This is perhaps not surprising in view of the weak van der Waals coupling between layers in bulk crystals. An important implication is an anisotropic order parameter in the bulk. Presumably, the CDW order along *z* melts at *T* just above the bulk *T*_C_=200 K, but the individual TiSe_2_ molecular layers remain in the (2 × 2) phase. The layers are, however, no longer phase locked, resulting in an overall (1 × 1 × 1) configuration on average. Nevertheless, the persistence of (2 × 2) of the individual layers can give rise to (2 × 2) band folding above bulk *T*_C_ as seen in Fig. 2b. Note that the single-layer *T*_C_=232 K determined here is for the layer sitting on a graphene-terminated SiC. It is not necessarily the same as that for a freestanding layer or a layer embedded in bulk TiSe_2_. The random interface potential caused by the lattice mismatch between graphene and TiSe_2_ could suppress the CDW *T*_C_ relative to the other cases. In our experiment on the single layer, evidence of band-folding disappears completely at *T* greater than the single-layer *T*_C_ (Fig. 2a). This is consistent with a single order parameter, as opposed to the bulk case.

Single-layer TiSe_2_ is an interesting candidate as a substitute of graphene or a complementary platform for building novel 2D electronics. Its natural gap is well suited for traditional semiconductor device architecture. The size of the gap is well matched to low-power designs and furthermore can be tuned by temperature and likely by other environmental effects. Single-layer TiSe_2_ is also an excellent prototypical system to unravel the long-standing mysteries and debates about the CDW transition in bulk TiSe_2_ and other related materials. For the single-layer case, the CDW phase is simply a band structure effect based on energy minimization. It is a more robust phase than the bulk case. The latter depends additionally on the layer stacking order, which melts at a lower temperature. This anisotropic order could be a common feature of layered CDW systems.

## Methods

### Film growth and characterization

Thin films of TiSe_2_ were grown *in situ* in the photoemission systems at beamlines 12.0.1 and 10.0.1, Advanced Light Sources, Lawrence Berkeley National Laboratory, where ARPES measurements were made. Substrates of 6H-SiC(0001) were degassed at 650 °C for several hours and then flash-annealed up to 1,300 °C for multiple cycles to form well-ordered bilayer graphene as verified by ARPES (ref. 14). High-purity Ti and Se were evaporated from an electron-beam evaporator and a Knudsen effusion cell, respectively, onto a substrate maintained at 220 °C. The growth process was monitored by a reflection high-energy electron diffraction system and the growth rate was controlled to be 30 min per single layer of TiSe_2_. Formation of the second layer of TiSe_2_ is evidenced by evolution of the band structure. The bulk TiSe_2_ samples were prepared by cleavage in the same vacuum chamber to expose a fresh surface. ARPES measurements were performed at a base pressure of ∼3 × 10^*−*11^ mbar. The system energy resolution was <20 meV, and the angular resolution was 0.2°. For band mapping along *k*_*z*_, a series of measurements was made with various photon energies in the range of 30–80 eV (ref. 19). Each sample orientation was precisely determined by constant-energy-contour mapping in k space to identify the high-symmetry points and the crystallographic directions.

### Theoretical calculation methods

First-principles calculations were performed using the Vienna *ab initio* package^20^,^21^,^22^ with the projector augmented wave method^23^,^24^. The monolayer system was simulated with a 17-Å vacuum region to suppress the interaction between adjacent layers. A plane wave energy cut-off of 320 eV and an 18 × 18 × 1 k-mesh were used for structure optimization. Using the generalized gradient approximation with the Perdew–Burke–Ernzerhof functional^25^, the equilibrium (1 × 1) lattice constant was found to be 3.538 Å for monolayer TiSe_2_, and the (2 × 2) CDW phase has a lower energy at *T*=0. The structure optimization was performed until the forces were reduced to below 0.005 eV Å^−1^. Once the atomic displacements were determined by the Perdew–Burke–Ernzerhof functional, a more accurate band structure for the fixed geometry was obtained by using the HSE functional^26^ including spin–orbit coupling. The HSE self-consistent calculations with 25% exact exchange were performed on a 12 × 12 × 1 (6 × 6 × 1) k-mesh for the normal (CDW) phase, and the band energy at an arbitrary k point was deduced by interpolating the Hamiltonian on the basis of maximally localized Wannier functions using the Wannier90 package^27^,^28^.

## Additional information

**How to cite this article:** Chen, P. *et al*. Charge density wave transition in single-layer titanium diselenide. *Nat. Commun*. 6:8943 doi: 10.1038/ncomms9943 (2015).
